# Musculus psoas major morphology - a novel predictor of mortality in elderly polytraumatized patients

**DOI:** 10.1186/s12873-023-00783-0

**Published:** 2023-02-07

**Authors:** Gregor Wollner, Valerie Weihs, Stephan Frenzel, Silke Aldrian, Lukas Leopold Negrin

**Affiliations:** Department of Orthopedics and Trauma Surgery, Medical University of Vienna, Vienna, Austria

**Keywords:** Polytrauma, CT-imaging, Musculus psoas major, Mortality, Elderly patients

## Abstract

**Introduction:**

Numerous papers in different fields have already shown that CT imaging of the Musculus Psoas Major (MPM) can be used to predict patient outcome. Unfortunately, most of the methods presented in the literature are very complex and not easy to perform in the clinic. Therefore, the objectives of the study were to introduce a novel and convenient method for measuring the MPM to trauma surgeons and to prove the association between MPM morphology and mortality in elderly polytraumatized patients.

**Material and methods:**

The retrospective outcome study was conducted at our level I trauma center. All patients admitted from 2006 to 2020 were included if they (1) presented with multiple injuries (≥2 body regions) and an Injury Severity Score (ISS) ≥16, (2) were at least 65 years of age, and (3) were diagnosed using a whole-body computed tomography. Subsequently, the ratios of short-axis to long-axis of both MPM were measured, and their mean value was evaluated as a candidate predictor of 31-day mortality.

**Results:**

Our study group consisted of 158 patients (63.3% male; median age, 76 years; median ISS, 25). In the survivors (55.7%), the mean MPM score was significantly higher compared to the fatalities (0.57 versus 0.48; *p* < 0.0001). Multivariate binary logistic regression analysis identified the MPM score as a protective predictor of 31 day-mortality (OR = 0.92, *p* < 0.001), whereas age (OR 1.08, *p* = 0.002 and ISS (OR 1.06, *p* = 0.006) revealed as significant risk factors for mortality. ROC statistics provided an AUC = 0.724 (*p* < 0.0001) and a cut-off level of 0,48 (sensitivity, 80.7%; specificity, 54.3%).

**Conclusion:**

The present study demonstrated that MPM score levels lower than 0.48 might be considered an additional tool to identify elderly patients at high risk of death following major trauma. In our opinion, the assessment of the MPM score is an easy, convenient, and intuitive method to gain additional information quickly after admission to the hospital that could be implemented without great effort into daily clinical practice.

## Introduction

It is well known that population aging leads to a drastic change in society, and therefore health care systems will face extraordinary challenges within the near future worldwide [1, 2]. It is generally expected that the proportion of the world’s population over 60 years will nearly double from 12 to 22% until 2050 [3]. Therefore, it is safe to assume that the number of elderly trauma victims will also rise. In central Europe, 20.2% of all documented polytraumatized patients are older than 65 years, and traumatic injuries are the fifth leading cause of death in this age class [4, 5]. Furthermore, it has been shown that elderly people (> 65 years of age) suffering a polytrauma have an increased risk of mortality [4–7]. Reported reasons are comorbidities, chronic intake of antiplatelet and anticoagulant medication, coagulopathy, acidosis, a low Glasgow Coma Scale (GCS), the presence of a large subdural hematoma, septic complications, and respiratory failure [5–7].

Whole-body computed tomography (WBCT) scans have a high sensitivity and are considered a standardized procedure for managing patients suffering multiple trauma in most centers [8]. Recently, computed tomography (CT) imaging of the Musculus Psoas Major (MPM) has been reported to have a predictive value in numerous different fields of medicine, including traumatology [9–12]. Whereas most publications presented a complex and time-consuming method for measuring and calculating the MPM, necessitating expertise and an elaborate image analysis program, Hanaoka et al. [13] used a quick and easy technique that was described by Inokuchi et al. [14] already in 2001. For calculating the cross-sectional area of the bilateral MPM, they digitally measured its short-axis and long-axis bilaterally at the level of the L3 vertebra using axial CT images [13]. Since the ratio of the short to the long axis provides information on the elliptic shape of the cross-sectional area, the authors assessed the left and right ratio and calculated their mean value, referred to as MPM score [13]. The aim of the present study was to investigate whether this score, which can be quickly and easily determined in the resuscitation room by treating clinicians, may identify elderly polytraumatized patients at a high risk of death.

## Material and methods

### Patients and study design

**Fig. 1:**
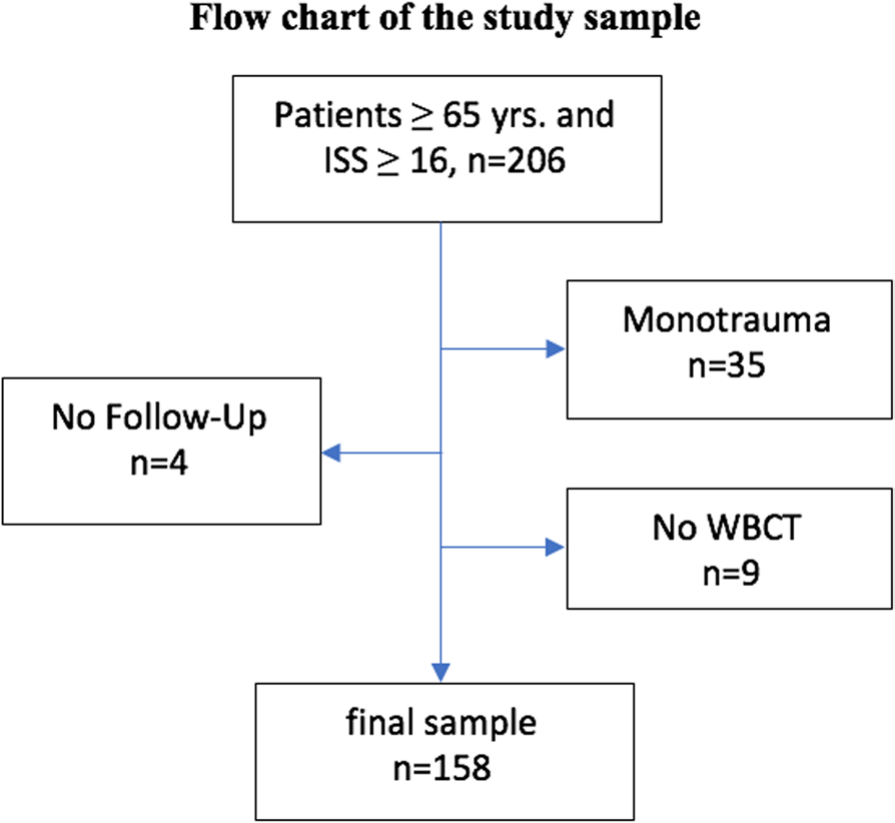
Flow chart of the study sample

This retrospective outcome study was approved by the Ethics Committee of the Medical University of Vienna (EK# 1942/2020) and complied with the guidelines of the Declaration of Helsinki. It is based on a data set, routinely gathered at a level I trauma center from 2006 to 2020 and consisting of all admissions to the resuscitation room. All patients included in this study (1) suffered multiple injuries (≥2 body regions), (2) presented with an Injury Severity Score (ISS) ≥16, (3) were at least 65 years of age at the time of admission, (4) and underwent a WBCT during their initial assessment. The flow chart of patient selection is presented in Fig. 1.

### Data collection

Parameters collected were baseline characteristics (age, gender), ISS, Glasgow Coma Scale (GCS), Abbreviated Injury Scale (AIS) of the different body regions, length of stay in the intensive care unit (ICU), number of ventilation days, and mechanism of injury. The primary outcome measure was all-cause 31-day mortality. All patients were followed up until death or discharge.

### Measurement of the MPM

**Fig. 2:**
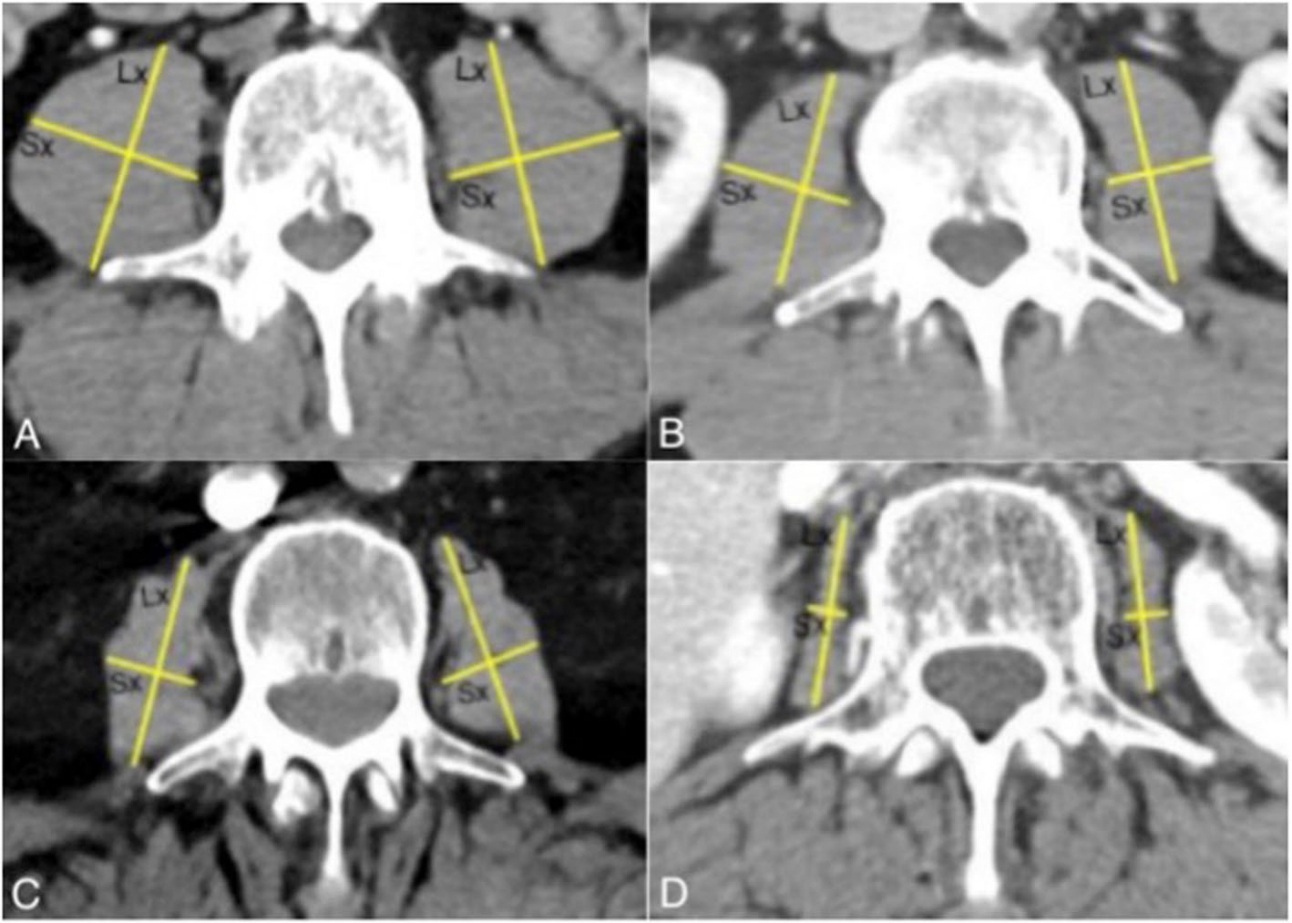
Measuring MPM in CT-images of axial views at the level of the L3 vertebra of a soft tissue window (Lx:Long axis, Sx: short axis; **A** = stadium 0, **B** = stadium 1, **C** = stadium 2, **D** = stadium 3)

Axial scans of WBCT were used to evaluate the morphology of the MPM. All scans were processed with PACS (Picture Archiving and Communication System), and one blinded investigator performed all measurements. A threshold of − 29 to + 150 Hounsfield units was applied to exclude the MPM from surrounding tissue following the literature [9, 13]. Just like Hanaoka et al. have done, we measured the length of the short-axis (Sx) and the length of the long-axis (Lx) of the MPM bilaterally at the level of the L3 vertebra on the first slice where both transverse processes were visible (Fig. 2) [13]. Subsequently, the left and right short-axis to long-axis ratios were assessed, and their mean value, the MPM score, varying from 0 to 1, was calculated. Similar as Inokuchi et al. had suggested, we defined four stadiums c [14].

Stadium 0: MPM score > 2/3

Stadium 1: 1/2 < MPM score ≤ 2/3

Stadium 2: 1/3 < MPM score ≤ 1/2

Stadium 3: 1/4 < MPM score ≤ 1/3

Measurements were performed in triplets, and the respective mean value was calculated.

### Statistical analysis

All statistical analyses were conducted using the SPSS® 26.0 software (SPSS Inc., Chicago, IL).

The Kolmogorov-Smirnow test was performed to assess the normal distribution of the parameters. Normally distributed parameters are expressed as mean ± standard deviation, whereas non-normally distributed parameters are presented as median and interquartile range (IQR) in round brackets. To compare two and four groups, we performed independent t-tests and one-way analyses of variance (ANOVA) respectively for normally distributed variables, whereas Mann-Whitney U-tests and Kruskal-Wallis tests were applied for non-normally distributed variables. In case of significant differences, Tukey’s method as a post hoc test for pairwise comparison was used for normally distributed samples. Frequency counts and percentages characterize categorical data. They were analyzed using the χ^2^ test. Since the outcome was dichotome (fatality yes/no) we conducted univariate and multivariate binary logistic regression analyses. The independent variables were age, ISS, and MPM score, the dependent variable was defined as the 31-day mortality. The Receiver Operator Characteristic (ROC) curve and the area under the curve (AUC) were calculated. The cut-off value was determined by the maximum sum of sensitivity and specificity [15]. Odds ratio (OR) and AUC were presented with a 95% confidence interval (CI) in square brackets.

The Spearman correlation coefficient was computed to test the association between selected parameters. Error bars and the stacked bar charts were established to visualize results. In general, a *p*-value < 0.05 was considered significant.

## Results

### Study population

One hundred and fifty-eight patients were included in the present study, of which 88 (55.7%) survived trauma for at least 31 days. There was no significant age gap between male and female patients (median, 75.9 (69.1-84.0) years versus 76.5 (71.5-83.3 years; *p* = 0.429). Table 1 lists all parameters collected in the present study overall and divided into four groups according to the stadium of the MPM. The pairwise comparison between the four mean MPM scores revealed significant differences for any possible combinations (*p* < 0.0001). Table 2 displays patients’ characteristics of survivors and fatalities. Whereas deceased patients had higher MPM scores than the surviving, the ISS did not differ significantly. A correlation of − 0.25 (*p* = 0.002) was calculated between age and MPM score (Table 3).

**Table 1:** Demographic and injury-related data

Variables	Overall	Stadium 0	Stadium 1	Stadium 2	Stadium 3	***p***-value
Patients, n	158 (100%)	23 (14.6%)	71 (44.9%)	59 (37.3%)	5 (3.2%)	
Male, n	100 (63.3%)	18 (78.3%)	53 (74.6%)	27 (45.8%)	2 (40.0%)	0.002**
Female, n	58 (36.7%)	5 (21.7%)	18 (25.4%)	32 (54.2%)	3 (60.0%)	
Age (years), median (IQR)	76.0 (70.0-83.6)	71.8 (68.0-80.0)	74.7 (68.0-82.0)	78.0 (72.9-86.0)	84.2 (76.0-86.2)	0.013*
MPM score, mean ± SD	0.53 ± 0.12	0.73 ± 0.07	0.57 ± 0.04	0.43 ± 0.04	0.29 ± 0.03	< 0.001**
Fatalities, n (%)	70 (44.3%)	2 (8.7%)	27 (38.0%)	37 (62.4%)	4 (80.0%)	< 0.001**
Stay at the ICU (days), median (IQR)	4.0 (0-16.0)	12.0 (0-24.0)	4.0 (0-18.0)	3.0 (0-12.0)	13.0 (2.0-23.0)	0.176
Ventilation time (days), median (IQR)	2.0 (0-10.0)	5.0 (0-17.0)	2.0 (0-10.0)	2.0 (0-6.0)	0 (0-11.0)	0.553
ISS, median (IQR)	25.0 (18.0-27.0)	21.0 (17.0-29.0)	25.0 (18.0-27.0)	25.0 (19.0-27.0)	24.0 (22.0-27.0)	0.809
AIS						
AIS head ≥3, n	107 (67.7%)	14 (60.9%)	47 (66.2%)	42 (71.2%)	4 (80.0%)	0.744
AIS abdomen ≥3, n	20 (12.7%)	4 (17.4%)	9 (12.7%)	6 (10.2%)	1 (20.0%)	0.791
AIS chest ≥3, n	68 (43.0%)	12 (52.2%)	32 (45.1%)	23 (39.0%)	1 (20%)	0.497
AIS face ≥3, n	11 (7.0%)	0	7 (9.9%)	4 (6.8%)	0	0.389
AIS extremities ≥3, n	25 (15.8%)	4 (17.4%)	12 (16.9%)	9 (15.3)	0	0.787
AIS external ≥3, n	0	0	0	0	0	–
GCS, median (IQR) (n, valid cases)	6.0 (3.0-15.0) (139)	12.0 (3.0-15.0) (17)	9.5 (3.0-15.0) (64)	3.5 (3.0-12.3) (54)	9.5 (4.5-13.8) (5)	0.091
Mechanism of injury						
Fall < 3 m, n	64 (40.5%)	7 (30.4%)	27 (38.0%)	27 (45.8%)	3 (60.0%)	
Fall > 3 m, n	6 (3.8%)	1 (4.3%)	2 (2.8%)	3 (5.1%)	0	
Traffic accident, n	69 (43.7%)	14 (60.9%)	30 (42.3%)	24 (40.7%)	1 (20.0%)	
Shot/stab, n	4 (2.5%)	0	3 (4.2%)	0	1 (20.0%)	
Other/unknown, n	15 (9.5%)	1 (4.3%)	9 (12.7%)	5 (8.5%	0

**Table 2:** Key values of Base line characteristics

Parameter	Survived *n* = 88 (55.7%)	Deceased *n* = 70 (44.3%)	*p*-value
Age years, median (IQR)	73.0 (69.1-81.0)	79.6 (73.0-87.0)	< 0.001^**^
Sex			
male, n (%)	60 (68.2)	40 (57.1)	0.153
female, n (%)	28 (31.8)	30 (42.9)	
MPM score, mean (±*SD*)	0.57 ± .12	0.48 ± .10	< 0.001^**^
ISS, median (IQR)	22.0 (18.0-27.0)	25 (20.0-28.0)	0.067

**Table 3:** Intercorrelation matrix, Spearman’s rank correlation (*n* = 158)

	Age	ISS	MPM
Age	1	−0.09	−0.025
ISS	−0.09	1	−0.08
MPM	−0.025	−0.08	1

### MPM as a predictor of 31-day mortality

**Fig. 3:**
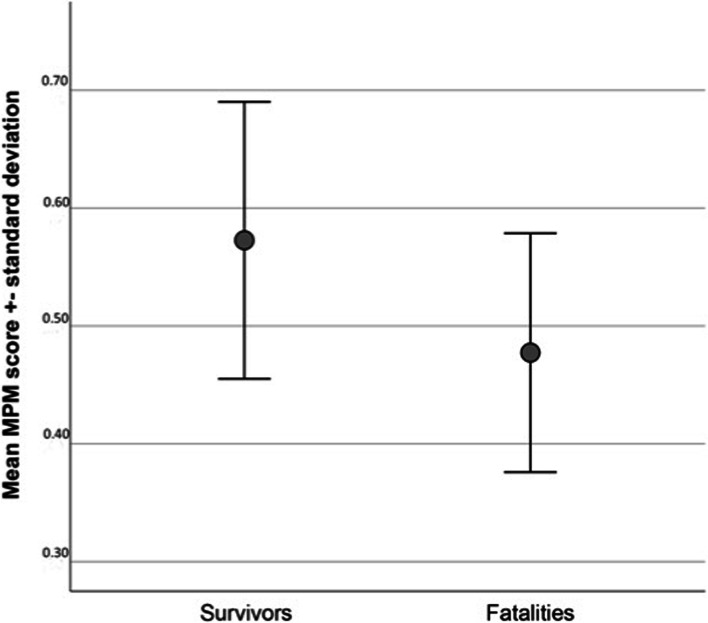
Mean MPM scores in survivors and fatalities

**Fig. 4:**
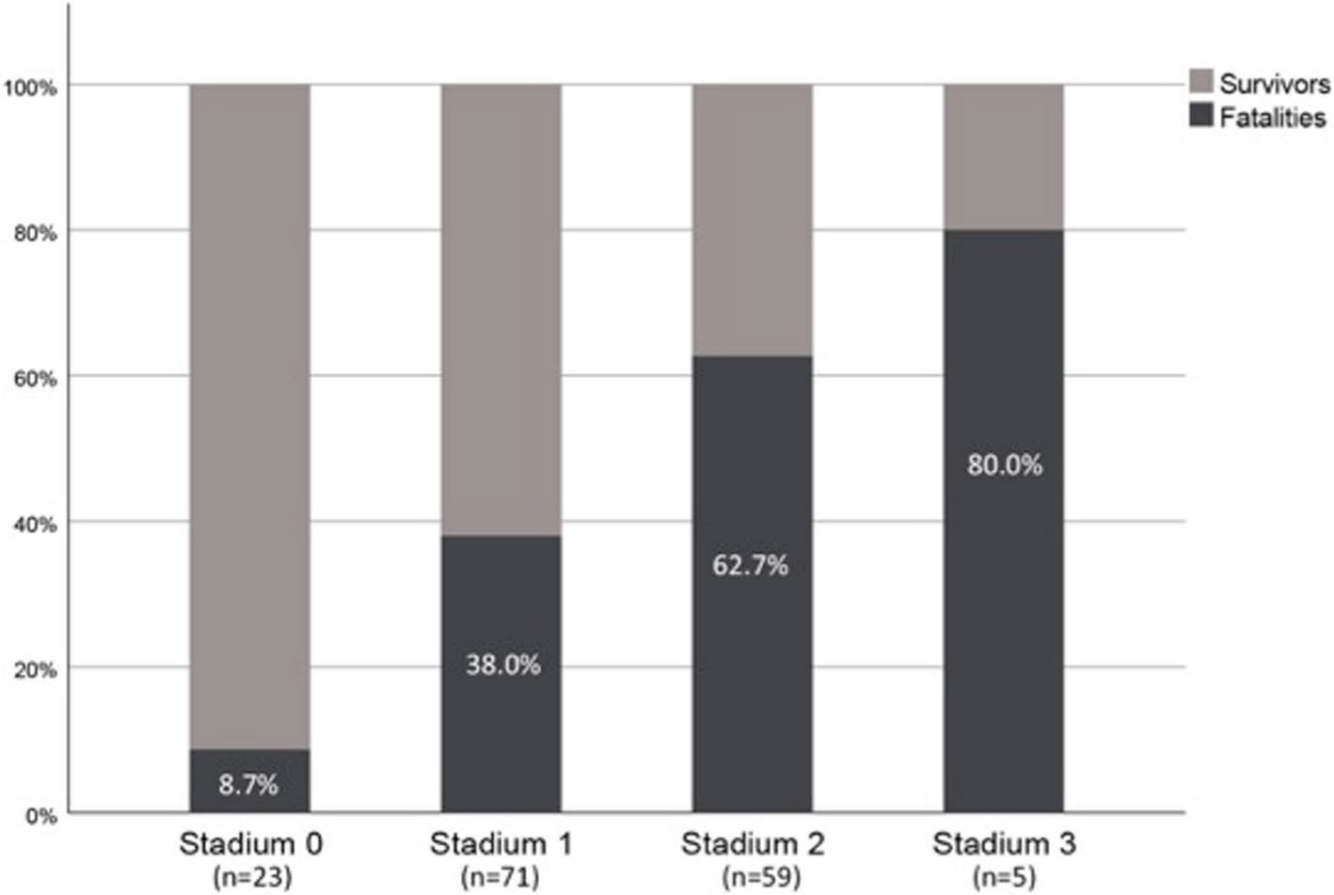
Survivors and fatalities in the four patient groups according to the MPM stadium

As displayed in Fig. 3, the mean MPM score was significantly higher in survivors than in fatalities (*p* < 0.001). Figure 4 presents the stacked bar chart of the survivors and fatalities in the four subgroups based on the stadium of the MPM, revealing an increase in the mortality rate from stadium 0 to 3 (*p* < 0.0001). Since the MPM score ranges from 0 to 1, the hundredfold MPM score defined the independent variable of the binary logistic regression analysis for the 31-day mortality. The odds ratio of 0.921 (95% CI [0.889-0.954], *p* < 0.0001) indicates that an increase of the MPM score by one-hundredth results in a decrease of the mortality rate by 7.9%. However, age and ISS had also be considered as predictors of 31-day mortality, resulting in an OR = 1.045 (95% CI [1.006-1085]; *p* = 0.023) and OR = 1.104 (95% CI, [1.028-1.184]; *p* = 0.006), respectively. To identify the factors that are independently related to 31-day mortality, we adjusted our model for age, sex and ISS.

According to Table 4 multivariate binary logistic regression analysis identified the MPM score as a protective predictor of 31 day-mortality, whereas age represent significant risk factors for mortality.

**Table 4:** Coefficients of predictors in predicting mortality (*n* = 158)

Predictor	*B*	*SE*	Wald (χ^2^) *df* = 1	*p*-value	OR	95% CI OR	
						LL	UL
Age	.075	.024	9.425	0.002^**^	1.078	1.027	1.130
ISS	.060	.022	7.626	0.006^**^	1.062	1.018	1.108
100 x MPM -score	−.079	.020	15.389	< 0.001^**^	.924	.889	.961
Constant	−3.436	2.354	2.131	0.144	.032		

Nagelkerke *R*^2^ = 33.3%

^**^p < 0.01

**Fig. 5:**
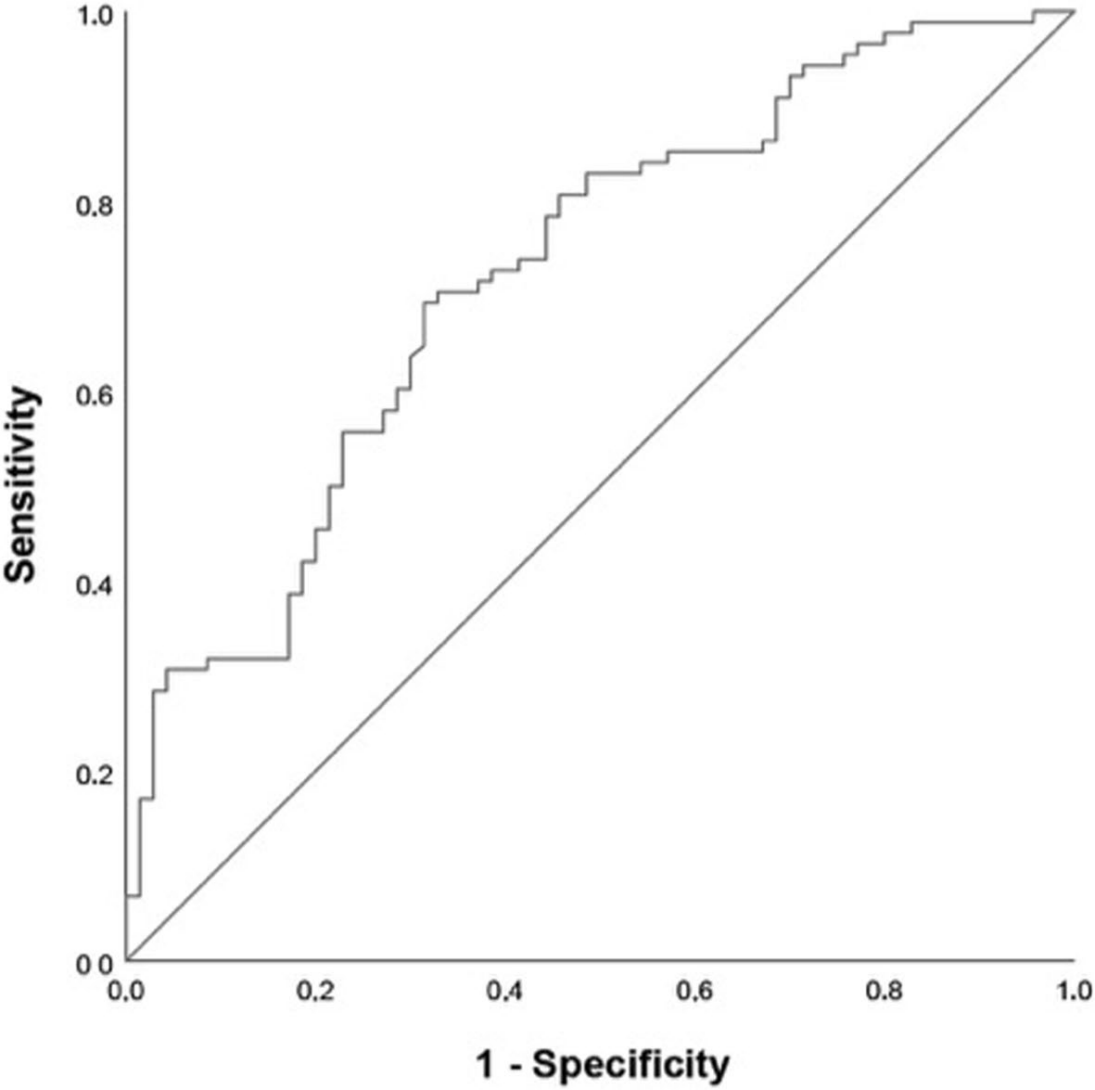
ROC curve of 31-day mortality and MPM score

To assess the diagnostic value of the MPM score as a predictor of the 31-day mortality, a ROC curve was generated (Fig. 5), indicating a fair test quality (AUC = 0.724, 95% CI [0.645-0.803], *p* < 0.001). 0.48 was determined as the cut-off value (sensitivity, 80.7%; specificity, 54.3%), with lower scores associated with mortality.

**Fig. 6:**
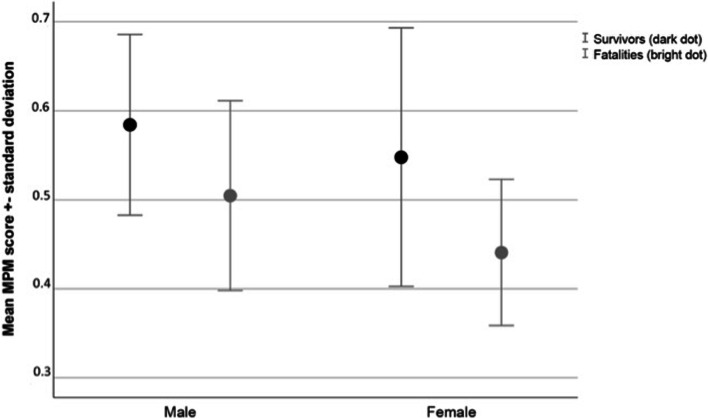
Mean MPM scores in survivors and fatalities separated by gender

Finally, we elucidated if gender-specific differences might have biased our results. Not surprisingly, the MPM score was significantly higher in males than in females (0.55 ± 0.11 versus 0.49 ± 0.13; *p* = 0.002). The mean MPM scores in survivors and fatalities separated by gender are presented in Fig. 6.

Multivariate binary logistic regression analyses provided an odds ratio of 0.924 (95% CI [0.881-0.968], *p* = 0.001) for males and of 0.961 (95% CI [0.863-0.973], *p* = 0.004) for females. An AUC of 0.690, 95% CI [0.582-0.799], *p* = 0.001) was calculated for males and an AUC of 0.732, 95% CI [0.601-0.863], *p* = 0.002) for females. 0.45 (sensitivity, 95.0%; specificity, 35.0%) and 0.48 (sensitivity, 60.7%; specificity, 66.7%) were determined as cut-off values for males and females respectively. We therefore see no need to provide gender-specific recommendations to clinicians.

## Discussion

Our study shows an innovative and promising way to reliably identify elderly polytrauma victims at high risk of mortality shortly after their admission to the trauma resuscitation room. Already viewing the shape of the MPM on CT scans raises the awareness of the trauma surgeon on duty for choosing the most proper treatment due to the patient’s overall situation. The main finding of our study is that low MPM scores, representing an MPM of elliptical shape with a short minor axis, may identify elderly polytrauma victims (≥ 65 years) at high risk of death. Using MPM scores lower than 0.48 as a predictor of 31-day mortality irrespective of gender is associated with a true positive rate of 80.7% and a false positive rate of 45.7%. Since this cut-off value is nearly identical to the threshold between stadiums 1 and 2, the lives of all patients presenting with stadiums 2 and 3 have to be considered in danger.

Frailty is a multifactorial syndrome that reflects multiple age-related declines of various physiological systems, finally leading to a decreased physiological reserve, loss of dynamic homeostasis, and increased vulnerability for morbidity and mortality [16, 17], posing unique challenges for clinical decision-making. Frailty has been shown to considerably impact trauma patient care, including hospital stay and discharge disposition from the hospital [18]. Sarcopenia, in turn, is a significant component of frailty [19], characterized by a progressive and generalized loss of skeletal muscle mass and a decline in strength and/or performance resulting in physical disability and death [20].

.Whereas the morphology of the MPM is considered valuable to assess sarcopenia by some authors [21, 22], other experts argue that it is not representative of all manifestations [23, 24]. According to the European Working Group on Sarcopenia in Older People (EWGSOP), further studies are needed to verify or reject the use of this method [25]. Nevertheless, CT-based assessment of the MPM has been reported as a predictive and straightforward indicator of morbidity and/or mortality in certain conditions (cirrhosis [11], hepatobiliary [26], colorectal [13], general [27], orthopedic [28], and vascular surgery [29]).

Three studies have already assessed the MPM morphology to define sarcopenia using CT imaging to predict mortality in trauma patients [10, 12, 30]. Nishimura et al. [10] evaluated 405 trauma victims with a minimum age of 65 years and an ISS ≥ 16. They defined the psoas muscle index (PMI) as the psoas muscle area at the third lumbar vertebra level divided by the body surface area separately for males and females. They were allocated to the non-sarcopenia group and the sarcopenia group, the latter combining all individuals with a PMI lower than the respective 25th percentile value. Group comparison revealed a higher 1-year mortality rate in the sarcopenia group, not reaching a significant difference. Yoo et al. [12] however, focused on 151 patients ≥45 years suffering blunt trauma (median ISS, 10.4). They defined the psoas index (PI) as the quotient of the sum of left and right psoas muscle area (assessed at the third lumbar vertebra level) and the square of the body height. 90-day mortality was significantly higher in sarcopenic patients presenting with a PI lower than the gender-specific 25th percentile value. Leeper et al. [30] generated the CAAST value (CT Abbreviated Assessment of Sarcopenia for Trauma), an adjusted measure of muscle mass, by measuring the psoas muscle cross-sectional area (at the third lumbar vertebra level) adjusted for height and weight using the 5th percentile for the CAAST value as the gender-specific cut-off. By evaluating 16,748 patients of at least 65 years, sarcopenia was identified as a strong predictor of 6-month post-discharge mortality for elderly adults [30].

.Unfortunately, each of the techniques for assessing the respective MPM parameter presented in these studies [10, 12, 30] necessitates a complex image analysis program that is usually unavailable in trauma centers. Contrarily, the method of Hanaoka et al. [13] and Inokuchi et al. [14] is intuitive and simple-to-use. They searched for a convenient marker of sarcopenia in colorectal cancer patients who underwent primary tumor resection with anastomosis and showed that MPM grading may predict postoperative complications. We adopted this method for polytraumatized patients because it is straightforward and quick to perform for two reasons. A WBCT is existent in the vast majority of trauma patients transferred to the resuscitation room, and lengths can be measured without additional soft- and hardware. Our approach is novel and valuable since we focused on the MPM score as the predictor of mortality in elderly trauma victims and did not make a detour by defining sarcopenia by MPM morphology, like the authors of the three comparison studies did in the first stage, evaluating the association between sarcopenia and mortality in the second stage. Moreover, their approach made it necessary to define arbitrary cut-off values separately in males and females. However, our gender-independent cut-off value results from a ROC analysis, correctly identifying 80.7% of elderly trauma victims at high risk of dying. Also, in our patients, a significant difference in MPM scores between survivors and fatalities could be revealed. Thus, we additionally performed binary logistic regression analyses and ROC statistics separated by gender. Since the cut-off value for males (0.45) was lower than the overall one (0.48), identifying a marginally smaller number of high-risk patients, and the cut-off value for females was even equally high, no necessity was demonstrated for two thresholds in our prediction model.

The allocation to the four subgroups according to the stadium of the MPM (Fig. 4) displays that especially trauma victims, whose cross-sectional MPM areas at the level of the L3 vertebra have an elliptical shape with a minor axis less than half of the major axis (stadium 2 and 3), are in a life-threatening condition, and thus require particular attention in the resuscitation room and later at the ICU. Whereas patients presenting with an almost circular-shaped MPM (stadium 0) can be immediately assigned to the subgroup with the lowest mortality rate (8.7%), for all others, their categorization to stadium 1 to 3 using the MPM score is mandatory as the respective mortality rates significantly differ (38.0, 62.7, and 80.0%).

Like the cross-sectional area of the MPM, the MPM score is a measure of muscle mass that has been reported to decrease with age [31]. In our patient population, an almost moderate negative Spearman correlation was observed between age and MPM score.

Quite unexpectedly, length of stay in the ICU and ventilation time did not vary within the groups. This finding is likely caused by the fact that we included all patients admitted to the resuscitation room in our study. Trauma victims who died during the initial assessment due to their severe injuries did not spend any time in the ICU, whereas patients who were transferred to the ICU after successful treatment stayed there for a long period.

## Limitations

Limitations of this study include the fact that data collected for a purpose other than the specific analysis have been evaluated in retrospect and that their acquisition occurred during an extended period. Due to the small number of patients, especially in stadium 3, statistical analysis of the different stadiums might be biased. Finally, the study population was limited to a single level I trauma center. Furthermore, the present study does not compare the technique for MPM-evaluation described by Hanaoka et al. with other techniques that require specific software.

## Conclusion

Since the assessment of the MPM score is a quick and easy technique to estimate the outcome of elderly patients not necessitating the acquisition of new equipment, it can be implemented as an additional tool in the clinical routine. We consider our paper a proof of concept that provides the basis for further research as part of prospective multicenter studies with a high number of patients.

## Data Availability

The analyzed dataset in this study is available from the first author on reasonable request.
